# Blood Lead Level and Renal Impairment among Adults: A Meta-Analysis

**DOI:** 10.3390/ijerph18084174

**Published:** 2021-04-15

**Authors:** Saruda Kuraeiad, Manas Kotepui

**Affiliations:** Medical Technology, School of Allied Health Sciences, Walailak University, Tha Sala, Nakhon Si Thammarat 80160, Thailand; saruda.ku@wu.ac.th

**Keywords:** blood lead level, renal insufficiency, renal impairment, BUN, creatinine

## Abstract

**Background:** The adult population in lead-related occupations or environmentally exposed to lead may be at risk for renal impairment and lead nephropathy. This meta-analysis aims to determine the impact of blood lead level (BLL) on renal function among middle-aged participants. **Methods:** Cross-sectional, longitudinal, or cohort studies that reported BLL and renal function tests among adult participants were retrieved from PubMed, Scopus, and ISI Web of Science. Relevant studies were included and assessed for quality using the Newcastle–Ottawa Scale (NOS). The pooled mean BLL of participants with a high BLL (≥30 µg/dL), moderate BLL (20–30 µg/dL), and low BLL (<20 µg/dL) was estimated using the random effects model. The pooled mean differences in BLL, blood urea nitrogen (BUN), creatinine, uric acid, and creatinine clearance between the exposed and non-exposed participants were estimated using the random effects model. Meta-regression was performed to demonstrate the association between the effect size (ES) of the pooled mean BLL and renal function. Heterogeneity among the included studies was assessed using the Cochrane Q and I^2^ statistics. Cochrane Q with a *p* value less than 0.05 and I^2^ more than 50% demonstrated substantial heterogeneity among the studies included. Publication bias was assessed using the funnel plot between the effect size and standard error of the effect size. **Results:** Out of 1657 articles, 43 were included in the meta-analysis. The meta-analysis demonstrated that the pooled mean BLL in the participants with a high BLL, moderate BLL, and low BLL was 42.41 µg/dL (95% confidence interval (CI): 42.14–42.67, I^2^: 99.1%), 22.18 µg/dL (95% CI: 21.68–22.68, I^2^: 60.4%), and 2.9 µg/dL (95% CI: 2.9–2.9, I^2^: 100%), respectively. The mean BLL of the exposed participants was higher than that of the non-exposed participants (weighted mean difference (WMD): 25.5, *p* < 0.0001, 95% CI: 18.59–32.45, I^2^: 99.8%, 17 studies). The mean BUN (WMD: 1.66, *p* < 0.0001, 95% CI: 0.76–2.55, I^2^: 76%, 10 studies) and mean creatinine (WMD: 0.05, *p* = 0.007, 95% CI: 0.01–0.08, I^2^: 76.8%, 15 studies) in the exposed participants were higher than those in the non-exposed participants. The mean creatinine clearance in the exposed participants was lower than that in the non-exposed participants (standard mean difference (SMD): −0.544, *p* = 0.03, 95% CI: −1.035–(−0.054), I^2^: 96.2%). The meta-regression demonstrated a significant positive effect of BLL on BUN (*p* = 0.022, coefficient: 0.75, constant: −3.7, 10 studies). **Conclusions:** BLL was observed to be associated with abnormal renal function test parameters, including high BUN, high creatinine, and low creatinine clearance. Moreover, BUN seemed to be the most valuable prognostic marker for lead-induced renal impairment. Therefore, regular checks for renal function among lead-exposed workers should be a priority and publicly promoted.

## 1. Background

Lead is a heavy metal and toxicant to the human body [1]. The most common sources of lead in lead-related occupations come from batteries, radiator manufacturing, lead refineries, paints, and ceramics [2]. In addition, lead is distributed in the environment as contaminated dust, in drinking water, and in soil where humans can be exposed through inhalation and ingestion [3]. When lead is absorbed into the blood, over 95% of it is bound to erythrocytes and distributed through the body [4].

Blood lead level (BLL) is widely used as a biomarker for lead exposure because it reflects the current exposure to lead [2]. Although the lead level in the environment has decreased for many years, diseases induced by lead have still been reported [3]. The benchmark based on the US National Health and Nutrition Examination Survey, which enrolled both children and adults, found a decrease in the mean BLL from 1.65 μg/dL in 1999–2000 to 0.84 μg/dL in 2013–2014 [5]. The US Department of Health and Human Services suggested that the BLL in adults should be below 10 μg/dL [4]. However, there is no defined safe value for lead, and a reference value for BLL has not been reported. In children, BLL at less than 10 µg/dL can cause intelligence deficits, anemia, and growth retardation [6,7]. In adults, lead can cause osteoporosis, hypertension, cardiovascular diseases, anemia, memory loss, and liver and renal impairment [8].

Evidence from epidemiological studies has demonstrated an association between BLL and lead nephropathy, where even BLL below 10 µg/dL can cause harmful effects on renal function [9,10]. A previous study demonstrated that BLL over 7.5 µg/dL presented a higher risk for renal impairment (odds ratio (OR) = 1.92, 95% confidence interval (CI): 1.18–3.10) and hyperuricemia [11]. Furthermore, investigators have shown that high BLL (>40 µg/dL) was associated with renal impairment among occupational workers [12,13,14,15]. Moreover, mortality from chronic kidney disease has increased among American lead workers [16]. Lead-induced lead nephropathy interferes with renal function in glomeruli and renal tubules, where the proximal tubule is reported to be highly sensitive to lead in short durations of high exposure, while chronic lead exposure induces glomerular sclerosis, and interstitial fibrosis induces irreversible renal injury [17]. A previous study suggested that frequent exposure to lead could induce the formation of lead-protein complexes deposited on the glomerulus and proximal tubules, which in turn reduces the glomerular filtration of urea and creatinine, leading to their retention in the plasma [17]. Studies have demonstrated that occupational lead workers, such as workers in battery plants, spray painters, and smelt lead workers, had significantly higher BLL than nonoccupational lead workers [7,13,17]. Therefore, lead-exposed workers have a higher risk for renal impairment [4]. Renal impairment can be assessed using renal function parameters, such as blood urea nitrogen (BUN), serum creatinine, uric acid, and creatinine clearance [18]. Studies by investigators on lead-exposed adult workers in Taiwan, South Korea, and Nigeria have demonstrated a decrease in creatinine clearance and an increase in BUN, serum creatinine, and uric acid among lead-exposed workers [14,15,19]. A study among South African battery plant workers showed an increase in serum creatinine and uric acid in lead-exposed workers, but they did not find any changes in BUN and creatinine clearance [13]. Nevertheless, some studies have demonstrated that all renal indicators were normal in lead-exposed workers [20].

A previous meta-analysis of BLL in Iranian lead workers demonstrated that the highest mean BLL was 72.58 µg/dL [95% CI: 26.06–119.10] among lead-zinc mine workers [21]. Moreover, the most recent meta-analysis investigating the association between BLL and cognitive function demonstrated that BLL was significantly higher in exposed than in non-exposed participants. In addition, an increase in BLL translated into a decline in cognitive abilities among exposed participants [8]. Although the meta-analysis of BLL related to works or the meta-analysis of BLL-related cognitive function have been investigated, to our knowledge, there is no meta-analysis investigating the association between BLL and renal function in adults currently. Therefore, the present study aimed to determine the impact of BLL on renal function among the adult population by determining the difference in BLL and the difference in renal function test parameters, including BUN, creatinine, creatinine clearance, and uric acid, among exposed and non-exposed participants. Moreover, meta-regression analysis was also performed to determine the association between BLL and renal function test parameters.

## 2. Methods

### 2.1. Protocol and Registration

The systematic review and meta-analysis followed the Preferred Reporting Items for Systematic Reviews and Meta-Analyses (PRISMA) guidelines [22]. The protocol of this study was registered at the International Prospective Register of Systematic Reviews (PROSPERO) with registration number CRD42021232630 [23].

### 2.2. Searches

The search terms in combination with the Boolean operator (“blood lead” OR “lead exposure” OR “lead toxicity” OR “lead poisoning”) AND (renal OR kidney) and (adult OR “middle-aged” OR worker) were used to retrieve potentially relevant studies in three databases: Medline, Scopus, and ISI Web of Science. The keywords “lead”, “renal”, and “middle-aged” were checked with the Medical Subject Heading (MeSH term) to identify the relevant search terms. The searches were performed on 15 January 2020. The search terms are provided in Table S1.

### 2.3. Eligibility Criteria

The inclusion criteria for this study included any cross-sectional, longitudinal, or cohort studies that reported on BLL and renal function among adult participants. The exclusion criteria excluded any studies with the following characteristics: animal models, case control studies on chronic kidney diseases or end-stage renal disease or patients with diabetes mellitus at enrollment, non-English language studies, studies with a small sample size (fewer than 10 participants) including case reports/case series/comments/errata/correspondences/short reports, in vitro studies, studies where the data on BLL or renal function tests could not be extracted, studies using the same data or participants for a different research question, and studies that measured co-exposure of lead and other metals.

### 2.4. Study Selection and Data Extraction

Any studies that met the eligibility criteria were selected by two independent authors (SK, MK). Any disagreement between the two authors in selecting the studies was resolved by discussion for consensus. Data extraction was also performed by two independent authors (SK, MK). The following data were extracted from the included studies: name of the first author, publication year, study site, year that the study was conducted, and participant data, including mean age, gender, BLL, BUN, creatinine, uric acid, creatinine clearance or estimated glomerular filtration rate (eGFR), and the status of renal impairment (if applicable). The data were extracted into a standardized pilot datasheet for further analysis.

### 2.5. Quality of the Included Studies (Risk of Bias)

The quality of the included studies was assessed using the Newcastle–Ottawa Scale (NOS) to assess the quality of non-randomized studies in meta-analyses [24]. The NOS scale assessing the quality of the included studies used the star system to judge the included studies on three main perspectives: selection of the study groups, comparability of the groups, and ascertainment of outcome of interest. Any studies rated with a score of at least eight indicated a “high quality” study, whereas any studies rated between 5–7 indicated a “moderate quality” study. NOS scores lower than 5 indicated a “low quality” study.

### 2.6. Study Outcomes

The primary outcome of this study was to explore the association between BLL and renal function. The secondary outcome was the pooled mean difference in BLL between the exposed and non-exposed participants. The third outcome was the pooled mean difference in BUN, creatinine, uric acid, and creatinine clearance between the exposed and non-exposed participants. The fourth outcome was the pooled mean BLL among participants with a high BLL (≥30 µg/dL), moderate BLL (20–30 µg/dL), and low BLL (<20 µg/dL). Cutoff values of <20 µg/dL, 20–30 µg/dL, and >30 µg/dL were used to indicate lead exposure among participants, as previously described by Lim et al. [25].

### 2.7. Statistical Analysis

The mean BLL reported in the included studies was used to analyze the pooled mean BLL among the exposed participants. However, as the non-exposed participants were a low-risk group and showed a low BLL, the mean BLL of non-exposed or controls was not estimated in this study. The median with rank or interquartile rank of BLL and renal parameters reported in the included studies was transformed into the mean and standard deviation (SD), as reported elsewhere [26]. The unit of BLL and renal parameters, including BUN, creatinine, and uric acid, used for analyses were µg/dL and mg/dL, respectively; hence, any studies that reported a different unit then had data converted to µg/dL of BLL and mg/dL of renal parameters using the calculator available online [27]. The pooled mean BLL and 95% confidence interval of the included studies were estimated using the random-effects model. The pooled mean differences in BLL, BUN, creatinine, and uric acid between the exposed and non-exposed participants were estimated using the random-effects model and presented as weighted mean differences (WMDs) with 95% CIs. WMD is the difference in means between the mean value in exposed and non-exposed participants. As the mean creatinine clearance was reported in the included studies in different units, the standard mean difference (SMD) was used to estimate the difference in mean creatinine clearance between exposed and non-exposed participants. Meta-regression was performed to demonstrate the association between the effect size (ES) or WMD of BLL and renal function test parameters, including BUN, creatinine, BUN/creatinine ratio, creatinine clearance, and uric acid. Heterogeneity among the included studies was assessed using the Cochrane Q and I^2^ statistics. Cochrane Q with a *p* value less than 0.05 and I^2^ more than 50% demonstrated substantial heterogeneity among the included studies [28]. Of the heterogeneity that existed, the random-effects model was used for estimating the pooled variables, and if heterogeneity did not exist, the fixed-effects model was used for estimating the pooled variables. Subgroup analysis of BLL was performed to demonstrate any differences among the groups of exposed participants. Publication bias among the included studies was assessed by visualizing the funnel plot asymmetry. If the funnel plot demonstrated an asymmetrical distribution, Egger’s test was used to confirm whether the asymmetrical distribution of the funnel plot was caused by the small-study effects. All analyses were performed using Stata Version 14.2 (StataCorp, College Station, TX, USA).

## 3. Results

### 3.1. Search Results

**Figure 1:**
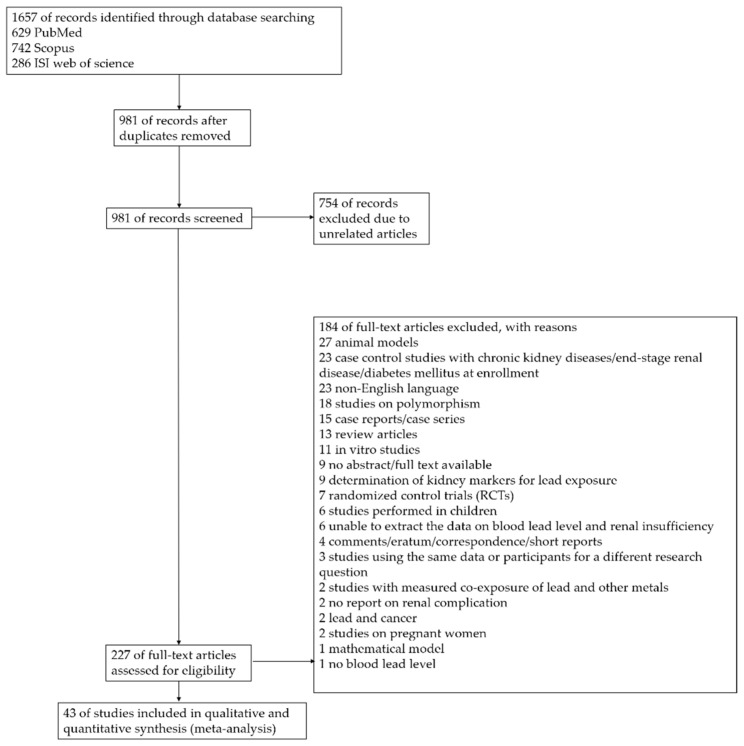
Study flow diagram.

Overall, 1657 articles were retrieved from the searches of three databases. After removing 676 duplicate articles, 981 articles were screened for potentially relevant articles through title and abstract screening. As a result, 754 articles were excluded due to their having no relevance to the present study. The full texts of the 227 articles that remained were examined according to the eligibility criteria, and 184 articles were excluded (Figure 1). Finally, 43 articles [3,4,7,11,13,15,16,17,19,20,25,29,30,31,32,33,34,35,36,37,38,39,40,41,42,43,44,45,46,47,48,49,50,51,52,53,54,55,56,57,58,59,60] met the study criteria and were included in the study.

### 3.2. Characteristics of the Included Studies

The 43 included studies were published between 1987 and 2020 and were conducted in 18 countries in 4 regions. Most of the studies were conducted in Asia (20/43, 46.5%), America (11/43, 25.6%), Europe (8/43, 18.6%), and Africa (4/43, 9.3%). In Asia, the studies were conducted in the Republic of Korea (5/20, 25%), India (4/20, 20%), China (4/20, 20%), and Taiwan (2/20, 10%), and the remaining 5 studies (25%) were from Thailand, Turkey, Iran, Japan, and Singapore. In America, the studies were conducted in the United States of America (9/11, 81.8%), and 2 studies (18.2%) were conducted in Brazil and Mexico. In Europe, the studies were conducted in Belgium (3/8, 37.5%) and Sweden (3/8, 37.5%), and 2 studies (25%) were conducted in the Netherlands and United Kingdom. In Africa, the studies were conducted in Nigeria (3/4, 75%) and South Africa (1/4, 25%). Most of the included studies were cross-sectional studies (32/43, 74.4%), and the rest (11/43, 25.6%) were cohort, longitudinal, or retrospective studies. Most of the studies determined BLL in exposed and non-exposed participants (18/43, 41.9%), population-based surveys (10/43, 23.2%), and BLL in only in exposed participants (15/43, 24.8%). The characteristics of the included studies are shown in Table 1.

### 3.3. Quality of the Included Studies

The quality of the included studies is shown in Table S2. Seventeen studies were high-quality studies, as BLL was reported in both the exposed and non-exposed participants. However, the rest of the included studies were low-quality studies, as they did not enroll a control group. Low-quality studies were included in the present study to analyze the pooled mean BLL.

### 3.4. Pooled Mean Blood Lead Level (BLL) among Exposed Participants

**Figure 2:**
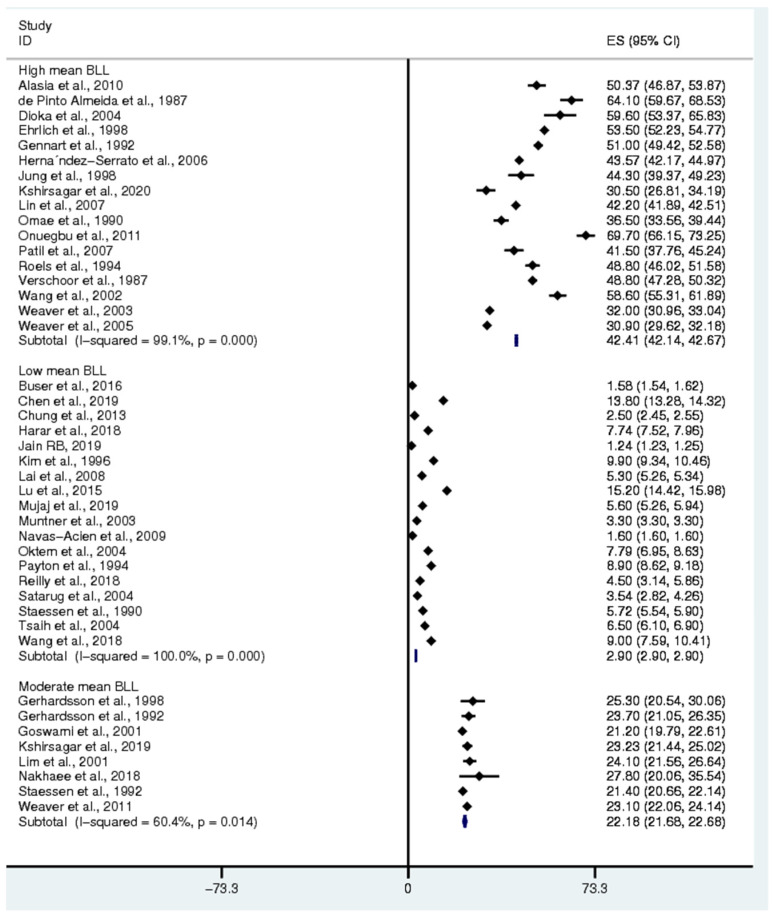
The mean BLL among participants. ES: Effect Size (mean BLL in ug/dL), CI: Confidence Interval (ug/dL), black diamond symbol: point estimate, solid line in the middle of the graph at 0: zero effect size.

The pooled mean BLL was estimated from all 43 studies [3,4,7,11,13,15,16,17,19,20,25,29,30,31,32,33,34,35,36,37,38,39,40,41,42,43,44,45,46,47,48,49,50,51,52,53,54,55,56,57,58,59,60] as all 43 studies reported the mean BLL of exposed participants (Figure 2). Among all the participants, the pooled mean BLL was sub-grouped into high mean BLL (BLL > 30 µg/dL), moderate mean BLL (BLL = 20–30 µg/dL), and low mean BLL (BLL < 20 µg/dL). Subgroup analysis demonstrated that the pooled mean BLL in the participants with a high mean BLL was 42.41 µg/dL (95% CI: 42.14–42.67, I^2^: 99.1%), whereas the pooled mean BLL in the participants with moderate and low mean BLL was 22.18 µg/dL (95% CI: 21.68–22.68, I^2^: 60.4%) and 2.9 µg/dL (95% CI: 2.9–2.9, I^2^: 100%), respectively. The characteristics of the exposed participants divided into three groups are shown in Table 2.

### 3.5. Pooled Mean Difference in BLL between Exposed and Control Participants

**Figure 3:**
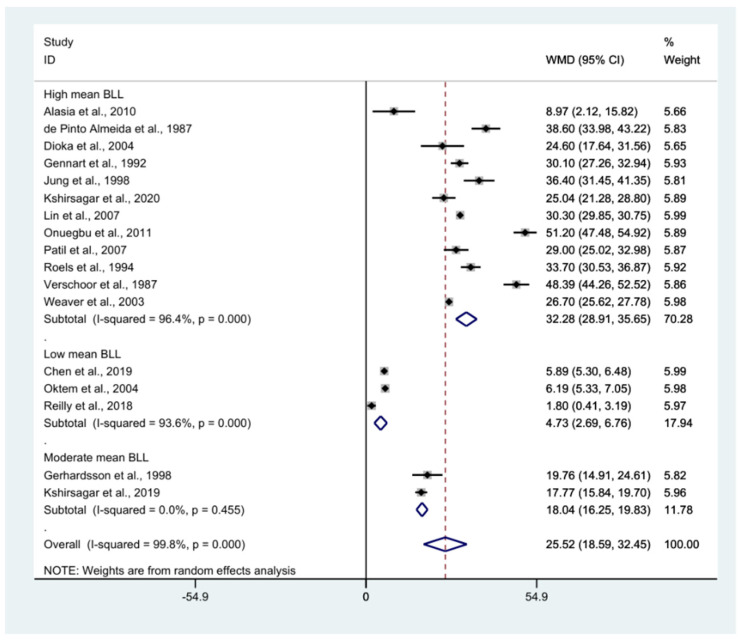
The mean difference in BLL between exposed and non-exposed participants. WMD: Weighted Mean Difference (µg/dL), % Weighted: the impact proportion of each study to the pooled effect, CI: Confidence Interval (µg/dL), Black diamond symbol: point estimate for each study, White diamond symbol: pooled WMD in each subgroup or all groups, Solid line in the middle of the graph at 0: no difference in WMD between the two groups, Dashed line: pooled WMD between the two groups.

The pooled mean difference in BLL between the exposed and non-exposed participants was estimated using the mean BLL from 17 studies [7,15,16,17,20,30,32,33,34,39,41,42,47,49,51,56,59] (Figure 3). Overall, the mean BLL of the exposed group was higher than that of the non-exposed participants (weighted mean difference: 25.5, *p* < 0.0001, 95% CI: 18.59–32.45, I^2^: 99.8%). Subgroup analysis demonstrated that the difference in BLL between exposed and non-exposed participants was larger for those with high mean BLL (weighted mean difference: 32.28, *p* < 0.0001, 95% CI: 28.91–35.65, I^2^: 96.4%), whereas the difference between exposed and non-exposed participants was smallest for those with a low mean BLL (weighted mean difference: 4.73, *p* < 0.0001, 95% CI: 2.69–6.76, I^2^: 93.6%).

### 3.6. BLL and Gender

**Figure 4:**
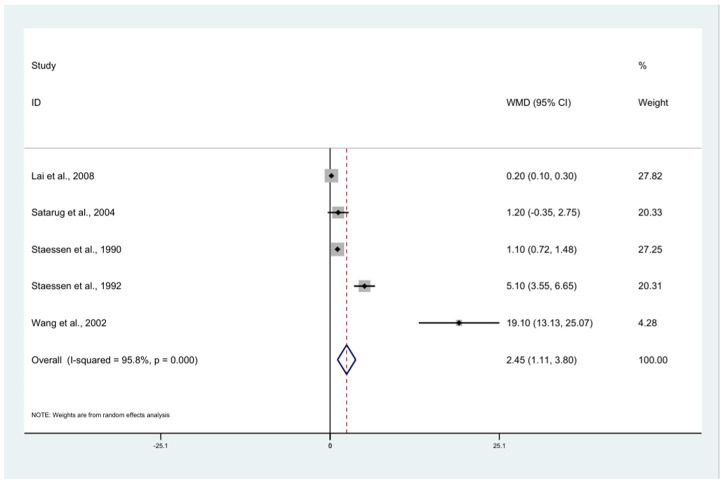
The mean difference in BLL between male and female participants. The mean BLL in the exposed males was higher than that in the female participants (weighted mean difference: 2.45, *p* < 0.0001, 95% CI: 1.11–3.80, I^2^: 95.8%) (white diamond symbol). WMD: Weighted Mean Difference (µg/dL), % Weighted: the impact proportion of each study to the pooled effect, CI: Confidence Interval (µg/dL), Black diamond symbol: point estimate for each study, White diamond symbol: pooled WMD in each subgroup or all groups, Solid line in the middle of the graph at 0: no difference in WMD between the two groups, Dashed line: pooled WMD between the two groups.

The pooled mean difference in BLL between the exposed men and women was estimated using five studies [11,19,52,53,54]. The results demonstrated that the mean BLL in the exposed males was higher than that in the female participants (weighted mean difference: 2.45, *p* < 0.0001, 95% CI: 1.11–3.80, I^2^: 95.8%) (Figure 4). Three studies [19,53,54] demonstrated a higher mean BLL in male participants than in female participants.

### 3.7. Renal Function Tests

**Figure 5:**
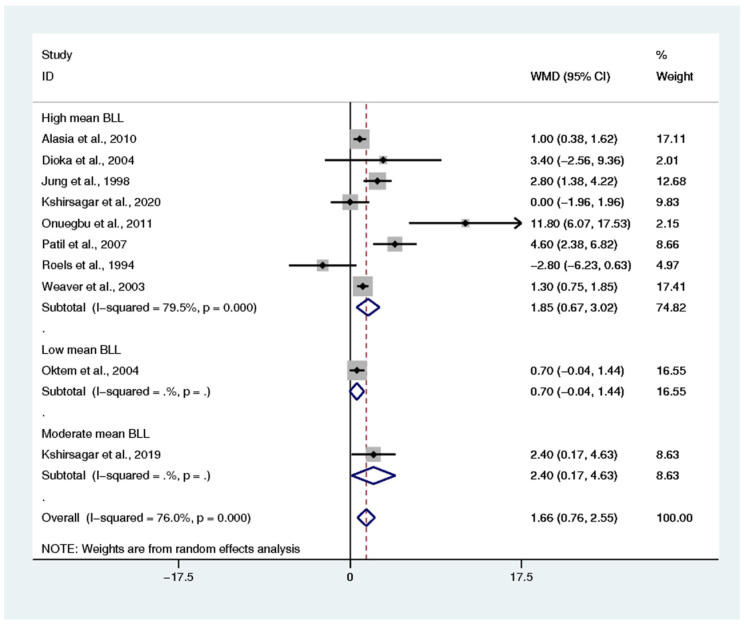
The mean difference in blood urea nitrogen (BUN) levels between exposed and non-exposed participants. WMD: Weighted Mean Difference (mg/dL), % Weighted: the impact proportion of each study to the pooled effect, CI: Confidence Interval (mg/dL), Black diamond symbol: point estimate for each study, White diamond symbol: pooled WMD in each subgroup or all groups, Solid line in the middle of the graph at 0: no difference in WMD between the two groups, Dashed line: pooled WMD between the two groups.

**Figure 6:**
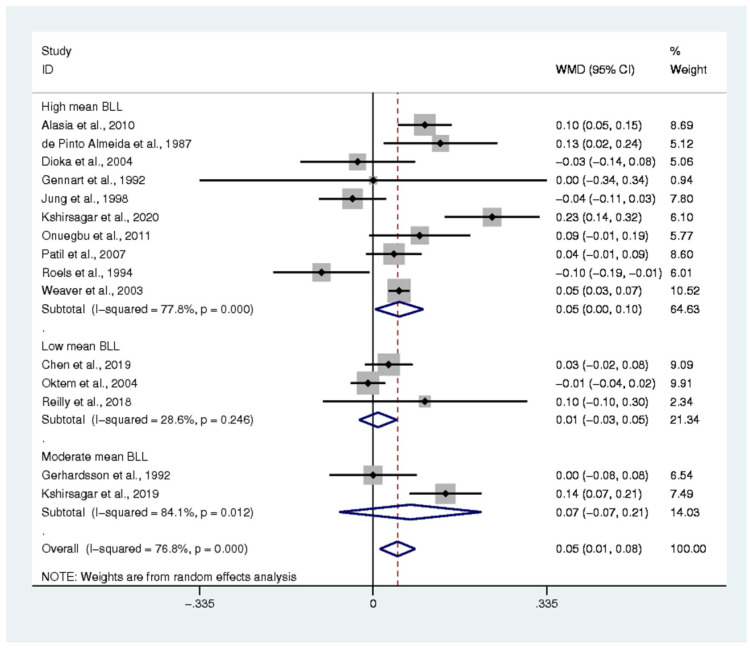
The mean difference in creatine levels between exposed and non-exposed participants. WMD: Weighted Mean Difference (mg/dL), % Weighted: the impact proportion of each study to the pooled effect, CI: Confidence Interval (mg/dL), Black diamond symbol: point estimate for each study, White diamond symbol: pooled WMD in each subgroup or all groups, Solid line in the middle of the graph at 0: no difference in WMD between the two groups, Dashed line: pooled WMD between two groups.

The difference in renal function parameters, including BUN, creatinine, uric acid, and creatinine clearance, of the exposed and non-exposed participants was estimated. The pooled mean difference in BUN between the two groups was estimated from 10 studies [7,15,17,20,32,39,41,47,49,59]. The results demonstrated that the mean BUN in the exposed group was higher than that in the non-exposed participants (weighted mean difference: 1.66, *p* < 0.0001, 95% CI: 0.76–2.55, I^2^: 76%) (Figure 5). The pooled mean difference in creatinine between the two groups was estimated from 15 studies [7,15,16,17,20,30,32,33,35,39,41,47,49,51,59]. The results demonstrated that the mean creatinine in the exposed participants was higher than that in the non-exposed participants (weighted mean difference: 0.05, *p*: 0.007, 95% CI: 0.01–0.08, I^2^: 76.8%) (Figure 6). The pooled mean difference in uric acid between the two groups was estimated from 9 studies [7,15,16,20,32,39,41,47,49]. The results demonstrated no difference in the mean uric acid of the exposed and non-exposed participants (weighted mean difference: 0.51, *p*: 0.061, 95% CI: −0.024–1.06, I^2^: 91.6%) (Supplementary Figure S1). The pooled mean difference in creatinine clearance between the two groups was estimated from 8 studies [15,20,30,33,47,51,56,59]. The results demonstrated that the mean creatinine clearance in the exposed participants was lower than that in the non-exposed participants (standard mean difference: −0.544, *p*: 0.03, 95% CI: −1.035–(−0.054), I^2^: 96.2%) (Supplementary Figure S2).

### 3.8. Renal Function Tests and BLL

**Figure 7:**
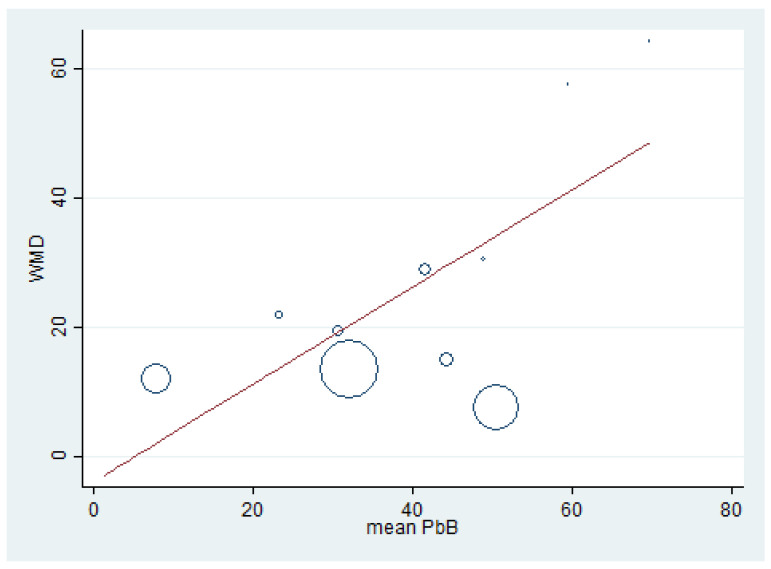
The meta-regression analysis of WMD (BUN) and mean BLL (µg/dL). WMD: Weighted Mean Difference, BUN: Blood Urea Nitrogen (mg/dL), PbB: Blood Lead (µg/dL).

**Figure 8:**
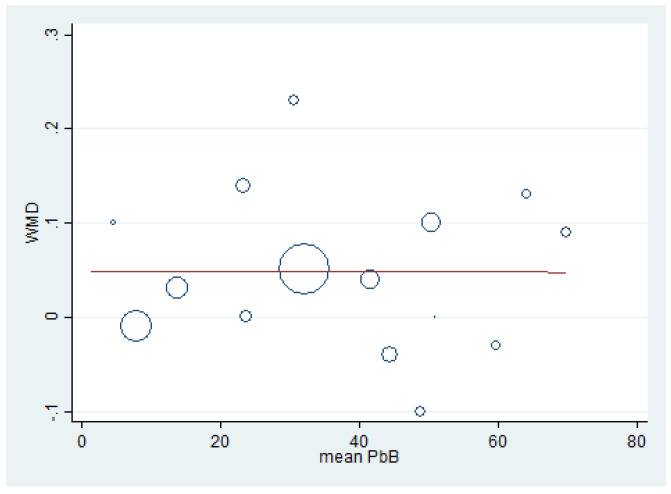
Meta-regression analysis of WMD (creatinine) and mean BLL (µg/dL). WMD: Weighted Mean Difference, PbB: Blood Lead (µg/dL).

**Figure 9:**
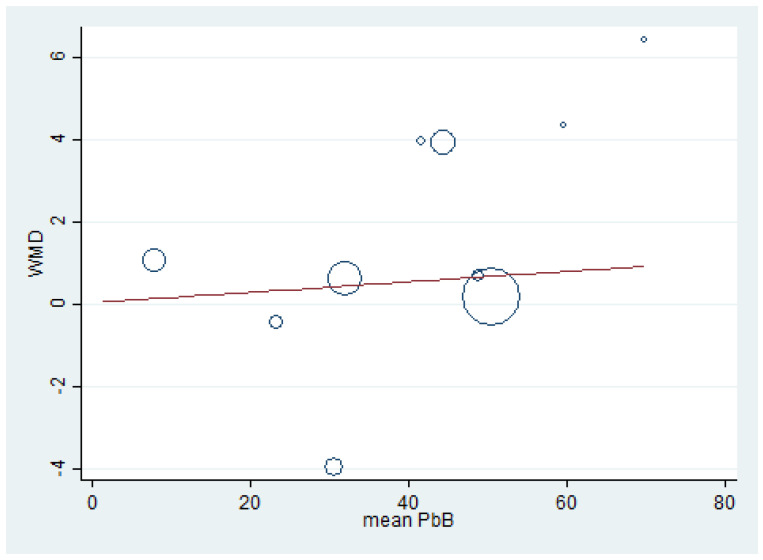
Meta-regression analysis of WMD and the BUN/creatinine ratio. WMD: Weighted Mean Difference, BUN: Blood Urea Nitrogen (mg/dL), PbB: Blood Lead (µg/dL).

Meta-regression analyses were performed to determine the association between the effect size (weighted mean difference, WMD) of renal function test parameters (dependent variable) and mean BLL (independent variable). The meta-regression of BUN (weighted mean difference) and mean BLL was performed using the data from 10 studies [7,15,17,20,32,39,41,47,49,59] because these studies reported the mean BLL and mean BUN. The results demonstrated a significant positive effect of BLL on BUN (weighted mean difference) (*p* = 0.022, coefficient: 0.75, constant: −3.7) (Figure 7). The meta-regression of creatinine (weighted mean difference) and mean BLL was performed using the data from 15 studies [7,15,16,17,20,30,32,33,35,39,41,47,49,51,59]. The results demonstrated a non-significant effect of mean BLL on creatinine level (weighted mean difference) (*p* = 0.989) (Figure 8). The meta-regression of mean BLL and the BUN/creatinine ratio (weighted mean difference) was performed using the data from 10 studies [7,15,17,20,32,39,41,47,49,59]. The results demonstrated a non-significant effect of mean BLL on the BUN/creatinine ratio (weighted mean difference) (*p* = 0.889, coefficient: 0.12, constant: 0.034) (Figure 9). No significant effect of mean BLL on creatinine clearance or uric acid was found (Supplementary Figures S3 and S4).

### 3.9. Publication Bias

**Figure 10:**
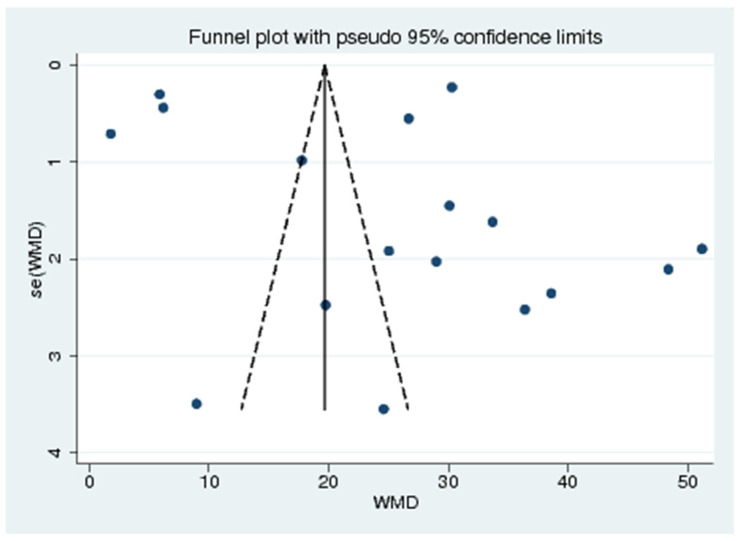
Funnel plot. WMD: Weighted Mean Difference, se (WMD): Standard Error (Weighted Mean Difference)

The funnel plot between the effect size (weighted mean difference) and standard error of the effect size demonstrated the likelihood of asymmetry (Figure 10). Therefore, Egger’s test was performed to confirm the funnel plot asymmetry. The results showed that no small-study effects among the included studies were found (*p* < 0.728), indicating no publication bias across the included studies.

## 4. Discussion

The present meta-analysis demonstrated that the mean BLL among participants with high BLL was 42.41 µg/dL, moderate BLL was 22.18 µg/dL, and low BLL was 2.90 µg/dL. The mean BLL was significantly higher in lead-exposed participants than in non-exposed participants for all 18 included studies. This finding was similar to a meta-analysis performed in Iran, which demonstrated high mean BLL in Iranian lead-exposed workers [21]. Moreover, the high difference in BLL seemed to be observed clearly among participants with high BLL compared to those with moderate or low BLL. Various studies supported this difference in the mean BLL among the two groups of participants. In Brazil, de Pinto Almeida et al. demonstrated that the mean BLL was 64.1 ± 16.3 µg/dL and 25.5 ± 4.4 µg/dL in primary lead smelting workers and in non-exposed participants, respectively [16]. In Germany, it was reported that the mean BLL was 40.6 (20.2–70.6) µg/dL in workers who were exposed to lead dust in an accumulator plant, whereas the mean BLL was 6.8 (4.8–10.6) µg/dL in the control group [12]. In South Africa, Ehrlich et al. reported that the mean BLL in battery factory workers was 53.5 ± 12.7 µg/dL [13]. In India, Patil et al. showed that the mean BLL in battery manufacturing workers, silver jewelry workers, spray painters, and controls was 53.63 ± 16.98, 48.56±7.39, 22.32 ± 8.87, and 12.52 ± 4.08 µg/dL, respectively [49]. In Nigeria, Alasia et al. showed that the mean BLL was 50.37 ± 24.58 µg/dL in lead-exposed workers and 41.40 ± 26.85 µg/dL in non-exposed participants [61]. Onuegbu et al. performed a study on automobile mechanics, battery repair workers, and petrol station attendants and demonstrated that the mean BLL was 69.7 ± 13.2 µg/dL in lead-exposed group and 18.5 ± 3.6 µg/dL in non-exposed participants [17]. Recently, a study in India also showed that the mean BLL was 30.5 ± 12.2 µg/dL in spray paint workers and 5.46 ± 2.58 µg/dL in the control group [7]. In South Korea, Jung et al. performed a study among workers who worked in secondary lead smelter, plastic stabilizer, and radiator manufacturing industries and showed that the mean BLL in highly exposed, moderately exposed, lowly exposed, and non-exposed participants was 74.6 ± 7.8, 46.5 ± 5.9, 24.3 ± 2.7, and 7.9 ± 1.4 µg/dL, respectively [39]. In Taiwan, a study by Hsiao et al. among lead battery factory workers showed that the mean BLL was 15.8 µg/dL and 11.6 µg/dL in males and females, respectively [62].

The pooled mean difference in BLL between the exposed males and females showed that the mean BLL in the exposed males was higher than that in the female participants. A significant difference in gender was clearly observed in a study by Staessen et al. [54] and Wang et al. [19]. However, a study by Lai et al. [11] demonstrated that BLL in exposed males was lower than that in female participants. The heterogeneity of the results between the studies might have been because males were more likely to be exposed to lead than females. Another possible explanation is that estrogen is higher in females than males; therefore, estrogen may increase lead distribution to the bone and slow the release of lead from the bone in women as well [63,64].

The present meta-analysis demonstrated the difference in the mean BUN, serum creatinine, and mean creatinine clearance in lead-exposed participants compared to non-exposed participants. In addition, the present meta-analysis showed that the mean BUN was significantly higher in lead-exposed participants than in non-exposed participants, especially in participants with high and moderate mean BLL. These results demonstrated that an increase in BLL could induce renal impairment among exposed participants. The difference in BUN was clearly observed in five included studies [15,17,39,49,59]. Nevertheless, some included studies demonstrated no difference in BUN between the two groups of participants [7,20,32] and caused heterogeneity among the studies included in the meta-analysis. A high mean BUN was also reported in lead battery workers and spray painters in India [7] and Taiwan [19], in a secondary lead refinery worker in South Korea [39] and Japan [65], and in lead workers in Nigeria [17] and India [49]. Moreover, Wang et al. demonstrated that every increment of 10 µg/dL BLL produced an increase of 0.62 mg/dL in BUN levels [19]. The increase in BUN might be caused by the reduction in renal plasma flow and the decrease in the glomerular filtration rate (GFR), leading to high accumulations of urea nitrogen in the plasma [66]. The meta-regression analysis between BLL and BUN demonstrated that the mean BLL was an independent factor affecting BUN levels. This result suggested that BUN is a sensitive marker of lead-induced renal impairment. In addition to the lead that affected the BUN levels, there were other factors, such as age, work duration, gender, and smoking habit [19].

The present meta-analysis showed that the mean creatinine was significantly higher in lead-exposed participants than in non-exposed participants, especially in participants with a high mean BLL. The higher mean creatinine among the exposed participants with a high mean BLL was clearly demonstrated in four studies [7,15,16,59]. High mean levels of creatinine were observed in various studies, such as the study by de Pinto Almeida et al., which studied Brazilian lead workers [16]; the studies of Onuegbu et al. [17] and Alasia et al. [61], which examined Nigerian lead workers; and the study of Kshirsagar Mandakini et al., which studied spray painters in India [7]. Nevertheless, five studies [17,32,33,39,49] demonstrated no difference in the mean creatinine between the two groups of participants. A study by Roels et al. [20] showed a lower mean creatinine in exposed participants than in non-exposed participants. Despite the high mean level of creatinine in the lead-exposed workers that was observed, the meta-regression showed no relationship between the mean BLL and creatinine level. Some previous studies reported similar findings to ours [32,39,49]. This might be because kidneys have millions of nephrons and have reserve capacity; therefore, the clinical manifestations of renal impairment would not be demonstrated until the nephrons were destroyed by more than 50% [49]. This indicated that serum creatinine was insufficiently sensitive for the early detection of renal impairment induced by lead. The non-association of BLL and creatinine might be due to factors related to creatinine balance, such as gender, age, weight, work duration, smoking habits, and alcohol consumption, which also affect serum creatinine [19].

Creatinine clearance has been widely used to determine GFR. It is commonly used in routine laboratory work for evaluating renal function. This study demonstrated that the mean creatinine clearance was significantly lower in lead-exposed workers than in non-exposed participants. This finding was observed in the studies by Alasia et al. [61], Gennart et al. [33], Weaver et al. [59], Chen et al. [30], and Reilly et al. [51]. Nevertheless, the meta-regression analyses did not show the relationship between the mean BLL and creatinine clearance. This finding is consistent with a study on lead-exposed workers in Japan [48], which indicated that a BLL less than 70 µg/dL might not affect the function of the glomeruli [54]. Furthermore, various confounding factors, such as ethnicity, age, gender, work duration, muscle mass, and protein intake, might influence creatinine clearance [19,44]. These confounding factors might, in part, affect the analysis of BLL and creatinine clearance.

Uric acid is the product of purine metabolism; moreover, it is derived from the degradation of a cell or nucleic acid within a cell, and elimination of uric acid occurs in the proximal tubule and distal tubule [67]. A previous study indicated that chronic lead exposure may interfere with the secretion of uric acid in the distal tubule, leading to hyperuricemia [68]. However, certain mechanisms of hyperuricemia induced by lead are still unclear. The present meta-analysis demonstrated no difference in the uric acid level between lead-exposed and non-exposed participants. Nevertheless, the exposed participants with a high BLL seemed to have a higher uric acid level, as demonstrated in four included studies [7,15,16,32]. In addition, a study by Kshirsagar et al. [41] demonstrated that exposed participants with a moderate BLL had a higher uric acid level than the control participants. Three studies demonstrated no difference in the serum uric acid level between the two groups of participants [20,39,47]. In addition, some previous studies contradicted our study [16,41,61]. These studies reported an increase in the uric acid level of lead-exposed workers who had a BLL greater than 60 µg/dL. Although the meta-analysis demonstrated a difference in the uric acid level between the two groups of participants, the meta-regression showed no association between BLL and mean uric acid. Therefore, the change in uric acid was insufficient as a sensitive marker to detect early renal impairment induced by lead exposure.

In addition to renal impairment induced by lead, lead exposure also increased the severity of underlying diseases, especially in susceptible populations with hypertension, diabetes mellitus, and chronic kidney disease [8]. Moreover, cadmium, mercury, and other heavy metals contaminating the environment and workplace may result in combined adverse effects on the human body. Therefore, protection from heavy metal exposure is crucial; for example, factory owners should provide occupational health educational programs to prevent workers from being poisoned by lead. In addition, lead exposure prevention should be implemented before, while and after the work is finished, for example, wearing personal protection devices, such as gloves, masks, and aprons, before starting to work, hand washing prior to eating, not smoking or eating in the workplace, and cleaning the body and mandatorily changing clothes before leaving the workplace to reduce the distribution of lead into the environment [65]. Although the removal and return of lead-exposed workers at 60 and 40 µg/dL, respectively, is used by the United States Occupational Safety and Health Administration (US OSHA) [69] and presumably by other countries, the results of the present study suggested that workers who have an excessive BLL of 30 µg/dL should be removed from their job and return to work when their BLL drops below 20 µg/dL.

## 5. Limitations

The present study had limitations. First, there were a limited number of included studies based on the eligibility criteria, which limited the study to adult or middle-aged participants. Second, the relationship between BLL and work duration was not assessed due to data unavailability among the included studies. Third, there are several factors that affect the progression of lead nephropathy in addition to lead, including individual susceptibility, race, and the pattern of lead exposure [39]. These might be the reasons for the heterogeneity among the included studies, where renal impairment was found to be related to lead exposure.

## 6. Conclusions

BLL was associated with abnormal renal function test parameters, including high BUN, high creatinine, and low creatinine clearance. Moreover, BUN seemed to be the most valuable prognostic marker for lead-induced renal impairment. Therefore, regular checks for renal function among lead-exposed workers should be important and publicly advocated for.

## Figures and Tables

**Table 1:** Characteristics of the included studies.

No. (Ref)	Author, Year	Study Area (Years of the Survey), Exposed Level	Study Design	Participants (Exposure and Control Groups)	Lead Exposure Group						Non-Exposed Group					
					Mean/ Median Age, Male (%)	BLL Levels (μg/dL), Duration of Exposure (Years)	BUN (mg/dL)	Creatinine (mg/dL), Creatinine Clearance (mL/min/1.72 m^2^)	Uric Acid (mg/dL)	Renal Insufficiency (n, %)	Age, Male (%)	BLL Levels (μg/dL), Duration of Exposure (Years)	BUN (mg/dL)	Creatinine (mg/dL), Creatinine Clearance (mL/min/1.72 m^2^)	Uric Acid (mg/dL)	Renal Insufficiency (n, %)
1. [15]	Alasia et al., 2010	Nigeria	Cross-sectional study	Study group (190); welding and metal (42), paint and pigment (38), radiator repairer (37), battery workers (37), petrol (36) Control group (80); hospital workers (80)	NS, 151/190 (79.5%)	50.37 ± 24.58, 11.9 ± 9.3	8.6 ± 2.3	1.0 ± 0.2, 98.9 ± 21.3	4.6 ± 1.2		58/80 (73)	41.40 ± 26.9, 8.0 ± 7.3	7.6 ± 2.4	0.9 ± 0.2, 108.2 ± 25.2	3.9 ± 1.1	
2. [29]	Buser et al., 2016	USA (2007–2008, 2009–2010, and 2011–2012)	Cross-sectional study	NHANES (4875)	NS, 2481/4875 (50.9%)	1.58 (1.49–1.67) or 1.58 ± 0.21		0.85 ± 0.00 (4785), 91.95 ± 0.58								
3. [30]	Chen et al., 2019	China	Cross-sectional study	Polluted area (174), non-exposed area (157)	Mean 58.7 (26–80), 52/164 (31.7%)	13.1 (8.36–20.6) or 13.8 ± 3.53		0.79 (0.7–0.95) or 0.81 ± 0.22, 94.7 (79.0–107.9) or 94.1 ± 8.34			56 (25–80), 59/157	7.44 (5.44–11.3) or 7.91 ± 1.71		0.77 (0.69–0.88) or 0.78 ± 0.21, 102.2 (91.2–112.7) or 102.1 ± 6.21		
4. [31]	Chung et al., 2013	The Republic of Korea (2007–2009)	Cross-sectional study	The Korea National Health and Nutrition Examination Survey (KNHANES) nationally representative survey (2005)	Mean 46 (20–87), male 49.8%	2.5 eGFR < 60 (83) 2.92 ± 0.13, eGFR ≥ 60 (1922) 2.53 ± 0.03		GFR: 90.0 ± 0.7								
5. [16]	de Pinto Almeida et al., 1987	Brazil	Cross-sectional study	Lead workers (52), reference (44)	44.9 ±9.54, NS	64.1 ±16.3		1.23 ± 0.34	6.6 ± 1.7	17/52	43.4 ± 8.9	25.5 ± 4.4		1.10 ± 0.20	4.7 ± 1.2	1/44
6. [32]	Dioka et al., 2004	Nigeria	Cross-sectional study	Exposed subjects (25); auto mechanics (18), battery chargers (5), welders (2) unexposed subjects (25); graduate students	39 ± 8.47, male 50/50 (100%)	59.6 ± 15.9	58.8 ± 13.6	1.12 ± 0.2	4.04 ± 1.39		Age matched	35 ± 7.9	55.4 ± 6.79	1.15 ± 0.2	2.58 ± 1.19	
7. [13]	Ehrlich et al., 1998	South Africa	Cross-sectional study	Battery making workforce (n = 382)	Mean 41.2 (8.3), NS	53.5 ± 12.7, 11.6 ± 6.8	5.6 ± 1.5	1.13 ± 0.18		BLL 23–50 µg/dL (160), 51–60 (115), 61–110 (101)						
8. [33]	Gennart et al., 1992	Belgium	Cross-sectional study	Exposed workers (98); lead acid battery factory control workers (85); the finishing department of the same factory, the maintenance department, the warehouse of a hospital and a chemical factory	37.7 ± 8.3, male 183/183 (100%)	51 ± 8, 10.6 ± 8.1		1.07 ± 1.16, 107 ± 1.22			38.8 ± 8.7	20.9 ± 11.1		1.07 ± 1.15, 110 ± 1.23		
9. [34]	Gerhardsson et al., 1998	Sweden	Cross-sectional study	Smelter workers (22); active workers (11), retired workers (11) referents (11); nearby machine-shop	NS, 22/22 (100%)	25.3 ± 11.4 Active workers 31.1 (7.67–49.7) or 29.8 ± 12.9, Retired workers 19.3 (11.2–33.2) or 20.7 ± 7.67						4.14 (2.07–7.05) or 4.35 ± 1.45				
10. [35]	Gerhardsson et al., 1992	Sweden	Cross-sectional study	Smelter workers (100); active workers (70), retired workers (30) referents (41); active truck assembly workers (31), retired truck assembly workers (10)	Active workers 37.4 ± 12–6), NS Retired workers 67.9 ± 47, NS	23.7 ± 13.5 Active workers 31.91 (4.97–47.45) or 29.1 ± 12.3, Retired workers 9.95 (3.32–20.93) or 11 ± 5.1 Duration of job: 19.8 ± 12.2 Active workers 14.3 ± 9.7, Retired workers (32.6 ± 6.3)		1.02 ± 0.26 Active workers 1.02 (0.75–1.32) or 1.03 ± 0.26, Retired workers 1.05 (0.71–1.23) or 1.01 ± 0.25 CRCL 102.4 ± 43.2; Active workers 105 (26–180) or 104 ± 44.5, Retired workers 87 (40–180) or 98.5 ± 40.4				5.54 ± 3.03 Active workers 4.14 (1.66 -12.4) or 5.59 ± 3.12, Retired workers 3.52 (2.28–12.2) or 5.38 ± 2.88		1.02 ± 0.22 Active workers 1.0 (0.84–1.15) or 1 ± 0.22, Retired workers 1.04 (0.89–1.32) or 1.07 ± 0.24 CRCL: Active workers 105 (26–180) µmol/dL, Retired workers 87 (40–180)		
11. [36]	Goswami et al., 2001	India	Cross-sectional study	372 Battery (63%), pigments (12.8%), rolled/extruded (7.7%), cable sheeting (4.5%), gas add (2.2%), others (9.9%)	36.2 ± 7.8, 372/372 (100%)	21.2 ± 13.9 Group A (185): 12.6 ± 3.9, Group B (63): 17.9 ± 2.1, Group C (99): 29.8 ± 9.6, Group D (25): 58.7 ± 11.3	Group A: 13 ± 8, Group B: 26 ± 7, Group C: 35 ± 13, Group D: 51 ± 12	1.1 ± 0.89 Group A: 0.9 ± 0.6, Group B: 1.2 ± 0.9, Group C: 1.3 ± 1.1, Group D: 1.5 ± 1.3 eGFR Group A: 141 ± 16, Group B: 86 ± 22, Group C: 55 ± 24, Group D: 33 ± 28		25 with advanced renal diseases						
12. [3]	Harar et al., 2018	Sweden (2007–2012)	Cohort study	4341 individuals enrolled and 2567 individuals subsequently followed up	Based line 57 ± 5.9, 1729/ 4341 (39.8%)	2.5 (0.15–25.8) or 7.74 ± 7.41		eGFR: based line (4272); 76 ± 14, followed up (2735); 70 ± 15		185 chronic kidney diseases						
13. [37]	Hernandez-Serrato et al., 2006	Mexico	Cross-sectional study	Exposed group (413): glazed pottery used, exposure occupation	37.27 ± 16.3, 156/413 (37.8%)	43.57 ± 14.5	33.17 ± 11.7	0.97 ± 0.23	6.47 ± 1.90	BLL ≥ 40 mg/dL (8/244) <40 mg/dL (4/169)						
14. [38]	Jain RB, 2019	USA (2003–2014)	Retrospective study	The data from National Health and Nutritional Examination Survey (NHANES): 9822 GF-1: 5710 GF-2: 3263 GF-3A: 563 GF-3B/4: 286	≥ 20, 5044/ 9822 (51.4%)	1.24 ± 0.32 Glomerular function (GF) GF-1: 1.05 (1.02–1.09) or 1.05 ± 0.21, GF-2: 1.42 (1.37–1.47) or 1.42 ± 0.2, GF-3A: 1.74 (1.63–1.87) or 1.75 ± 0.22, GF-3B/4: 1.87 (1.70–2.05) or 1.87 ± 0.23										
15. [39]	Jung et al., 1998	Republic of Korea	Cross-sectional study	Lead exposed workers (75): secondary lead smelter industry (27), plastic stabilizer industry (18), radiator manufacturing industry (30) control group (64): male office workers	41.5 ± 7.67, 75/75 (100%) Highly exposed (21): 43.6 ± 8.3, Moderately exposed (20): 42.3 ± 8.6, Slightly exposed (34): 39.7 ± 6.4	44.3 ± 21.8 Highly exposed: 74.6 ± 7.8, moderately exposed: 46.5 ± 5.9, slightly exposed: 24.3 ± 2.7 Duration of employed: 8.27 ± 4.29 Highly exposed: 8.5 ± 3.8, moderately exposed: 8.3 ± 6.2, slightly exposed: 8.1 ± 3.2	15.8 ± 4.54 Highly exposed: 18 ± 5.5, moderately exposed: 15.6 ± 3.9, slightly exposed: 14.6 ± 3.8	0.86 ± 0.19 Highly exposed: 0.9 ± 0.2, moderately exposed: 0.8 ± 0.1, slightly exposed: 0.8 ± 0.2	5.41 ± 1.43 Highly exposed: 6 ± 1.5, moderately exposed: 5.1 ± 1.1, slightly exposed: 5.2 ± 1.5	Highly exposed (2)	44.2 ± 8.6 (64)	7.9 ± 1.4, duration of employed: 8.1 ± 2.4	13 ± 4	0.9 ± 0.2	5.6 ± 1.5	1
16. [40]	Kim et al., 1996	USA (1979–1992)	Retrospective study	459 men randomly selected from the Normative Aging Study	56.9 ± 8.3, 459/459 (100%)	9.9 ± 6.1		1.22 (0.9–1.8) or 1.29 ± 0.33								
17. [7]	Kshirsagar et al., 2020	India	Cross-sectional study	Spray painters (42), normal healthy subjects (50)	Range 20–50, NS	30.5 ± 12.2	20.5 ± 4.78	1.21 ± 0.26	6.6 ± 2		20–50	5.46 ± 2.58	20.5 ± 4.78	0.98 ± 0.17	5.41 ± 1.03	
18. [41]	Kshirsagar et al., 2019	India (2018)	Cross-sectional study	Silver jewelry workers (42) control group (50)	Range 20–60, NS	23.23 ± 5.91	22.9 ± 5.93	1.12 ± 0.17	6.39 ± 1.18		20–60	5.46 ± 2.58	20.5 ± 4.78	0.98 ± 0.17	5.41 ± 1.03	
19. [11]	Lai et al., 2008	Taiwan	Cross-sectional study	2565 residents: aboriginals (1318), nonaboriginals (1247)	> 40, NS	5.3 ± 1.2 Male (1008): 5.3 ± 1.2, 5.6 ± 1.4), female (1557): 5.3 ± 1.1, 5.4 ± 1.2	Male (15.4 ± 4.3, 15.5 ± 4.6), female (14.9 ± 4.5, 15.7 ± 5.6)	1.1 ± 0.28 Male (1.2 ± 0.3, 1.1 ± 0.4), female (1.0 ± 0.2, 1.0 ± 0.5)	Male (6.9 ± 1.8, 8.6 ± 2.1), female (5.8 ± 1.8, 7.0 ± 1.9)	Aboriginals (153), Nonaboriginals (87)						
20. [25]	Lim et al., 2001	Singapore	Cross-sectional study	Workers from a factory producing polyvinyl chloride (PVC) stabilizers using lead ingots as raw materials (55)	35.73 ± 9.59, 55/55 (100%)	24.1 ± 9.6 <20 μg/dL (18), 20–30 μg/dL (23), > 30 μg/dL (14)			CRCL: (120.9 ± 14.9)	2 participants with CRCL < 90						
21. [42]	Lin et al., 2007	China	Cross-sectional study	Exposed group (135): one storage battery plant control group (143): mechanics	28.7 ± 6.6, NS	42.2 ± 1.86, 5.8 ± 4.4					27.0 ± 8.5	11.9 ± 1.96				
22. [43]	Lu et al., 2015	China (2013)	Cross-sectional study	Participants who live in a region of China with heavy metal pollution (1447)	46.68 ± 15.1, NS	15.2 ± 15.1	4.47 ± 3.49	CRCL: 76.78 ± 70.44		BLL 0–100 µg/L (669), ≥ 100 µg/L (778)						
23. [44]	Mujaj et al., 2019	USA (2015–2017)	Cross-sectional study	Newly hired workers at s at battery manufacturing and lead recycling plants in the USA (447)	BLL <3.0 (147): 28.8 ± 9.5), BLL 3.1–6.3 (152): 30.4 ± 11.4), BLL ≥ 6.3 (148): 27.3 ± 5.3 Male %: NS	5.6 ± 3.62 BLL < 3.0: 1.66 (1.3–2.5) or 1.78 ± 0.4, 3.1–6.3: 4.63 (3.9–5.7) or 4.72 ± 0.56, ≥ 6.3: 10.48 (7.9–12.25) or 10.3 ± 1.27		BLL < 3.0 µg/dL (0.97 ± 0.12), 3.1–6.3 µg/dL (0.99 ± 0.14), ≥6.3 µg/dL (0.96 ± 0.13) eGFR: BLL <3.0 µg/dL (105.4 ± 14.5), 3.1–6.3 µg/dL (102.6 ± 16.0), ≥ 6.3 µg/dL (107.7 ± 14.8)		BLL <3.0 µg/dL (147), 3.1–6.3 µg/dL (152), ≥ 6.3 µg/dL (148)						
24. [45]	Muntner et al., 2003	USA (1988–1994)	Retrospective study	Normotension by the National Center for Health statistics (10,398)	≥20, 4991/ 10,398 (48%)	3.30 ± 0.10		1.05 ± 0.004 eGFR: 115 ± 0.7		0.7–1.6 µg/dL (114), 1.7–2.8 (166), 2.9–4.6 µg/dL (229), 4.7–52.9 µg/dL (270) CKD (114)						
25. [4]	Nakhaee et al., 2018	Iran (2017)	Case-cohort study	Exposed group: healthy adults with chronic lead exposure (BLL > 10 μg/dL) (100), healthy individuals with BLL < 10 μg/dL (100)	45.8 ± 11.8, 184/200 (92%)	All group: 27.77 ± 39.45 BLL > 10 μg/dL (51.36 ± 44.72), BLL < 10 μg/dL (4.17 ± 1.97)	BLL > 10 μg/dL (34.0, 27.0–221.0), BLL < 10 μg/dL (30.0, 27.0–36.0)	BLL > 10 μg/dL (0.9, 0.8–1.0), BLL < 10 μg/dL (0.8, 0.7–0.9)								
26. [46]	Navas-Acien et al., 2009	USA (1999–2006)	Retrospective study	National Health and Nutrition Examination Survey (14,778): reduced eGFR (1668), normal GFR (13,110)	Reduced eGFR: 67.6 ± 0.5, 640/ 1668 (38.4%) Normal GFR: 44.7 ± 0.3, 6660/ 13,110 (50.8%)	1.6 ± 0.27 Reduced eGFR (<60): 2.06 (1.98–2.15) or 2.06 ± 0.21, normal GFR: 1.54 (1.50–1.57) or 1.54 ± 0.21 BLL: < 1.1 (147), 1.1–1.6 (274), 0.6–2.4 (468), > 2.4 (779)										
27. [47]	Oktem et al., 2004	Turkey	Cross-sectional study	Auto repairers (79), healthy control (71)	17.3 ± 1.0, NS	7.79 ± 3.81 BLL; 3.4–4.9 µg/dL (14): 4.11 ± 0.43, 5–9.9 µg/dL (51): 7.08 ± 1.38, 10–25 µg/dL (14): 14.04 ± 4.59	12.8 ± 2.3 BLL; 3.4–4.9 µg/dL (14): 12.5 ± 2.5, 5–9.9 µg/dL (51): 12.9 ± 2.2, 10–25 µg/dL (14): 13.1 ± 2.6	0.82 ± 0.08 BLL; 3.4–4.9 µg/dL (14): 0.83 ± 0.09, 5–9.9 µg/dL (51): 0.81 ± 0.08, 10–25 µg/dL (14): 0.84 ± 0.10 GFR: 147 ± 16.1 BLL; 3.4–4.9 µg/dL (14): 147 ± 17.9, 5–9.9 µg/dL (51): 149 ± 15.6, 10–25 µg/dL (14): 139 ± 14.5	5.6 ± 1.1 BLL; 3.4–4.9 µg/dL (14): 5.7 ± 0.9, 5–9.9 µg/dL (51): 5.5 ± 1.1, 10–25 µg/dL (14): 6.0 ± 1.1 GFR: 147 ± 16.1		17.0 ± 1.1	1.60 ± 0.80	12.1 ± 2.3	0.83 ± 0.12 GFR: 146 ± 18.5	5.9 ± 1.4	
28. [48]	Omae et al., 1990	Japan (1985)	Cross-sectional study	Lead exposed workers (165): duration of exposed > 10 years (20), duration of exposed < 10 (134)	18.4–57.3, NS	36.5 (6–73) or 36.5 ± 19.3 0–19 (21), 20–29 (39), 30–39 (34), 40–49 (36), 50–59 (25), ≥ 60 (10) Duration of exposed: > 10 years: 43.7 (23–73), Duration of exposed < 10: 36.2 (6–73)		0–19 (1 ± 1.13), 20–29 (0.96 ± 1.11), 30–39 (0.96 ± 1.14), 40–49 (0.95 ± 1.13), 50–59 (0.93 ± 1.10), ≥ 60 (0.97 ± 1.12) CRCL: 0–19 (99.3 ± 1.12), 20–29 (105.4 ± 1.13), 30–39 (104.5 ± 1.11), 40–49 (105.3 ± 1.14), 50–59 (110.1 ± 1.12), ≥ 60 (102.2 ± 1.18)								
29. [17]	Onuegbu et al., 2011	Nigeria	Cross-sectional study	Exposed workers (53): automobile mechanics (23), battery repair workers (11), petrol station attendants (19) Control (42)	30.8 ± 7.8, 53/53 (100%)	69.7 ± 13.2 automobile mechanics (68.8 ± 14.8), battery repair workers (75.5 ± 10.0), petrol station attendants (67.4 ± 12.4)	65 ± 14.8 automobile mechanics (69 ± 14.7), battery repair workers (55.4 ± 13.6), petrol station attendants (65.6 ± 13.6)	1.1 ± 0.32 automobile mechanics (1.09 ± 0.04), battery repair workers (1 ± 0.17), petrol station attendants (1.18 ± 0.32)			30.1 ± 1.2, 42/42	18.5 ± 3.6	53.2 ± 13.6	1.01 ± 0.15		
30. [49]	Patil et al., 2007	India	Cross-sectional study	All exposed group (90) Battery manufacturing industries (30), silver jewelry (30) workers, spray painters (30) control group (35)	20–40 years, 90/90 (100%)	41.5 ± 18.1 Battery manufacturing industries (53.6 ± 17, silver jewelry (48.6 ± 7.39) workers, spray painters (22.3 ± 8.87)	25.7 ± 9.59 Battery manufacturing industries (30.4 ± 11), silver jewelry (20 ± 5.84) workers, spray painters (26.7 ± 8.34)	0.85 ± 0.19 Battery manufacturing industries (0.83 ± 0.15), silver jewelry (0.83±0.20) workers, spray painters (0.88±0.22)	4.96 ± 1.26 Battery manufacturing industries (5.92 ± 0.95), silver jewelry (4.07 ± 1.01) workers, spray painters (4.90±1.10)		20–40 years, 35/35	12.52 ±4.08	25.12 ±5.73	0.81 ± 0.11	5.57 ± 0.97	
31. [50]	Payton et al., 1994	USA (1988–1991)	Cross-sectional study	Men participating in the Normative Aging Study (744)	64 ± 7.4, NS	8.9 ± 3.9		1.3 ± 0.2 CRCL: 88.2 ± 22, eGFR: 71 ± 18.4								
32. [51]	Reilly et al., 2018	USA	Cross-sectional study	Smelter-working resident (52) control residents (290)	55.8 ± 10.5, NS	4.5 ± 5 Duration of residence (14.1 ± 12.2)		1.3 ± 0.67 eGFR: 85.2 ± 26.5			43 ± 14.1	2.7 ± 2.5 Duration of residence (11.5 ± 11.9)		1.2 ± 0.66 eGFR: 96 ± 24.2		
33. [20]	Roels et al., 1994	Belgium	Cross-sectional study	Workforce of a large lead smelter (47) control group (55): the same workplace but never directly occupationally exposed to lead	42.3 ± 8.1, NS	46.6 (34.2–67.9) or 48.8 ± 9.74, 15.9 ± 6.8	29.7 (15.9–50.3) or 31.4 ± 9.93	0.91 (0.69–1.07) or 0.9 ± 0.23, 123.5 (97–177) or 130.3 ± 23.1	5.1 (3.3–8.2) or 5.43 ± 1.44		43.0 ± 9.1	13.9 (6.3–26.1) or 15.1 ± 5.73	32.4 (23.3–48.6) or 34.2 ± 7.31	0.97 (0.78–1.28) or 1 ± 0.25, 114.2 (81–156) or 116.4 ± 21.66	5.4 (3.8–8.1) or 5.68 ± 1.27	
34. [52]	Satarug et al., 2004	Thailand	Cross-sectional study	Students, factory workers, teachers, and laborers (118)	37.5 ± 8.8, 53/118 (44.9%)	3.54 ± 3.99 Male (53): 4.2 ± 5.4, female (65): 3.0 ± 2.2	Male 12.6 ± 3.4, female 11.0 ± 2.5	Male 0.94 ± 0.12, female 0.66 ± 0.10								
35. [53]	Staessen et al., 1990	United Kingdom (1982)	Cross-sectional study	Civil servants (531)	47.7 ± 5.77, 398/531 (75%)	5.72 ± 2.1 Male (398): 6.0 ± 2.1, female (133): 4.9 ± 1.9		Male 9.7 ± 2.6, female 7.8 ± 1.1								
36. [54]	Staessen et al., 1992	Belgium (1985–1989)	Prospective population-based Study	Exposed group (2327): the Malmo Diet and Cancer Study (MDCS-CC), prospective population-based study (MDCS)	48 ± 16, 965/ 2327 (41.5%)	21.4 ± 18.1 Male 11.4 (2.3–72.5) or 24.4 ± 20.3, female 7.5 (1.7–60.3) or 19.3 ± 16.9		Male 1.24 (0.7–4.64, female 1.05 (0.58–2.71) CRCL: Male 93 ± 30, female 480 ± 25								
37. [55]	Tsaih et al., 2004	USA	Cohort study	The Normative Aging Study (NAS)	Baseline (448): 66 ± 6.6, NS	Baseline (427): 6.5 ± 4.2, follow-up 4.5 ± 2.5		Baseline (448): 1.1 ± 0.4, follow-up 1.25 ± 0.2								
38. [56]	Verschoor et al., 1987	Netherlands	Cross-sectional study	155 lead workers (155): lead battery plants 1 (36), lead battery plants 2 (52), lead battery plants 3 (9), plastic stabilizer production plant (58) control workers (126): nonlead plants, insulation materials (60), production of drainpipes (56), plant producing concrete (10)	30–51, NS	Exposed group (148): 47.5 (33.8–66.5) or 48.8 ± 9.45 B plant 1: 50.15 (37.5–66.7), B plant 2: 45.4 (24.7–66.9), B plant 3: 65.9 (46.2–94.3), stab plant: 45.6 (34.2–60.7) BLL < 20.7 µmol/L (125), BLL 20.7–62.2 (113), BLL > 62.2 (27)	56.6 ± 14.1 BLL < 20.7 (56.6 ± 14.7), BLL 20.7–62.2 (56.6 ± 13.6), BLL > 62.2 (56.6 ± 13.6)	0.96 ± 0.16 BLL < 20.7 (125): 0.96 ± 0.16, BLL 20.7–62.2 (113): 0.96 ± 0.15, BLL > 62.2 (27): 0.92 ± 0.16 Relative CRCL: 0.17 ± 0.09	6.34 ± 1.4 BLL < 20.7 (6.29 ± 1.34), BLL 20.7–62.2 (6.42 ± 1.38), BLL > 62.2 (6.27 ± 1.78) Relative CRCL: 0.17 ± 0.09		30–51 years	0.40 (0.27–0.58) or 0.4 ± 0.22		Relative CRCL: 0.17 ± 0.08		
39. [19]	Wang et al., 2002	Taiwan	Cross-sectional study	Lead battery workers (229)	40 ± 14.7, 120/229 (52.4%)	58.6 ± 25.4 Male: 67.7 ± 28.2, female: 48.6 ± 17.0 BLL < 60 µg/dL (134), BLL > 60 µg/dL (95) Work duration: 8.24 ± 8.25 Male: 4.6 (0.2–35) or 11.1 ± 10.1, female: 2.7 (0.2–17) or 5.65 ± 4.87	BLL < 60 (14.37 ± 0.35), BLL > 60 (16.65 ± 0.43)	BLL < 60 (1.04 ± 0.01)), BLL > 60 (1.05 ± 0.02) Abnormal creatinine BLL < 60 (18), BLL > 60 (23)	BLL < 60 (5.66 ± 0.12), BLL > 60 (6.09 ± 0.15)							
40. [57]	Wang et al., 2018	China (2012)	Cross-sectional study	Lead exposure paint workers	31.7 ± 7.74, 706/747 (94.5%)	9.0 ± 6.0 (70) BLL positive (70)				Renal dysfunction (93), BLL positive and renal dysfunction (19/70), BLL negative and renal dysfunction (74/751)						
41. [58]	Weaver et al., 2011	Republic of Korea (2004–2005)	Cohort study	Current and former workers employed at 26 lead-using facilities (712)	47.6 ± 7.9, 563/712 (79%)	23.1 ± 14.1 Duration of exposed: 13.1 ± 7.3		0.87 ± 0.15 eGFR: 97.4 ± 19.2 CRCL: 111.1 ± 30.7								
42. [59]	Weaver et al., 2003	Republic of Korea (1997–1999)	Cohort study	Current and former lead workers (803): lead battery, lead oxide, lead crystal, radiator manufacture, and secondary lead smelting controls (135)	40.4 ± 10.1, 639/803 (79.6%)	32.0 ± 15.0 Duration of job: 8.2 ± 6.5	14.4 ± 3.7	0.90 ± 0.16 CRCL: 94.7 ± 20.7			34.5 ± 9.1, 124/135	5.3 ± 1.8	13.1 ± 2.9	0.91 ± 0.10 CRCL: 108.4 ± 19.4		
43. [60]	Weaver et al., 2005	Republic of Korea (1999–2001)	Cohort study	Workers from 26 plants that produced lead batteries, lead oxide, lead crystal, or radiators or secondary lead smelters (652)	43.3 ± 9.8, 503/652 (77.2%)	30.9 ± 16.7	14.4 ± 3.9	0.87 ± 0.15	109.2 ± 34.8							

Ref; reference number; BLL, blood lead level; BUN, blood urea nitrogen; CRCL, creatinine clearance; eGFR, estimated glomerular filtration rate; NS, not specified; KNHANES, The Korea Nation Health and Nutrition Examination Survey; PVC, Polyvinyl chloride.

**Table 2:** Sources of lead contamination among exposed participants.

High mean BLL (>30 µg/dL)	Sources of contamination: welding and metal, paint and pigment, radiator repair, petrol, auto mechanic, battery makers and chargers, glazed pottery, plastic stabilizer industry, radiator manufacturing industry, storage battery plant, automobile mechanic, petrol station, silver jewelry, lead battery plants, production plant, lead oxide, and lead crystal
Moderate mean BLL (20–30 µg/dL)	Sources of contamination: smelting, batteries, pigment, extruded materials, cable sheeting, gas add, silver jewelry, PVC-producing factory, stabilizers using lead ingots, lead-using facilities
Low mean BLL (<20 µg/dL)	Sources of contamination: polluted areas, heavy metal pollution, battery manufacturing and lead recycling plants, auto repair, smelting factory

## Data Availability

The dataset used and analyzed during the current study is available from the corresponding author upon reasonable request.

## Supplementary Materials

The following are available online at https://www.mdpi.com/article/10.3390/ijerph18084174/s1. Supplementary Figure S1. The mean difference in uric acid levels between exposed and control participants, Supplementary Figure S2. The mean difference in creatinine clearance between exposed and control participants, Supplementary Figure S3. The meta-regression analysis of WMD and creatinine clearance, Supplementary Figure S4. The meta-regression analysis of WMD and uric acid, Supplementary Tables, Table S1. Search terms, Table S2. Quality of the included studies, PRISMA Checklist S1.

## Abbreviation

BLL	Blood lead level
BUN	Blood urea nitrogen
CI	Confidence Interval
CRCL	Creatinine clearance
eGFR	estimated Glomerular Filtration Rate
GF	Glomerular function
KNHANES	The Korea Nation Health and Nutrition Examination Survey
NS	Not specified
PbB	Blood lead
PVC	Polyvinyl chloride
Ref	Reference number
WMD	Weighted Mean Difference
mg/dL	milligrams per deciliter
μg/dL	micrograms per deciliter
