# Supplementary material for: Blood Lead Level and Renal Impairment among Adults: A Meta-Analysis
Source: Int J Environ Res Public Health. 2021 Apr 15;18(8):4174. doi: 10.3390/ijerph18084174 (PMC8071292; doi:10.3390/ijerph18084174)
Supplement: Supplementary file 1 [file ijerph-18-04174-s001.zip › ijerph-1156141(XML)/Supplementary Figure S1-4.pdf]

Blood Lead Level and Renal Impairment among Adults: A Meta-Analysis

Saruda Kuraeiad [1], Manas Kotepui [1*]

[1] Medical Technology, School of Allied Health Sciences, Walailak University, Tha Sala, Nakhon Si Thammarat, Thailand

Authors' Email Addresses:

[*]**Corresponding Author**: Manas Kotepui; manaskote@gmail.com

Saruda Kuraeiad; saruda.ku@wu.ac.th

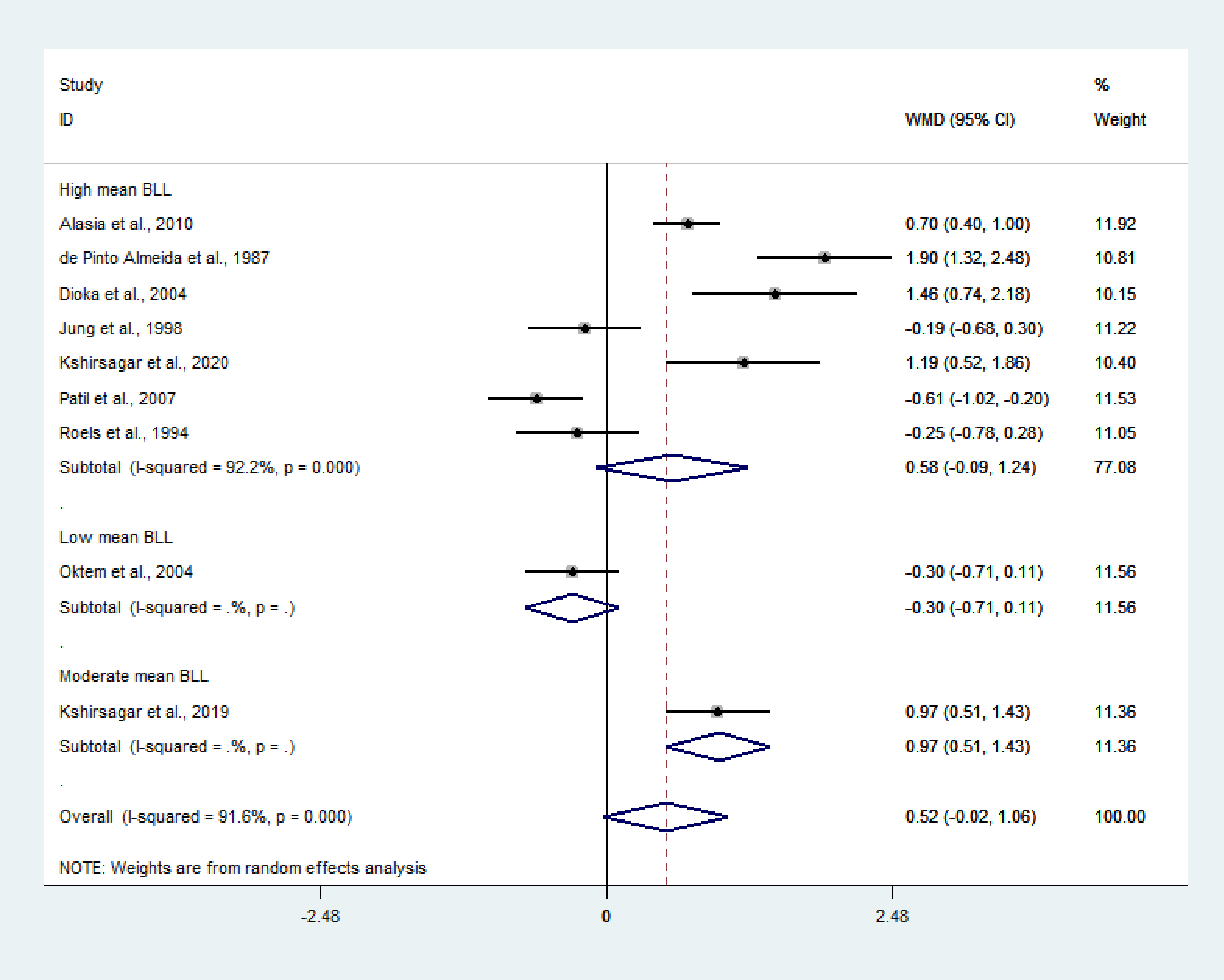

Supplementary figure 1. The difference in the mean uric acid of the exposed and non-exposed participants. WMD: Weighted Mean Difference (µg/dL), % Weighted: the impact proportion of each study to the pooled effect, CI: Confidence Interval (µg/dL), Black diamond symbol: point estimate for each study, White diamond symbol: pooled WMD in each subgroup or all groups, Solid line in the middle of the graph at 0: no difference in WMD between the two groups, Dashed line: pooled WMD between the two groups

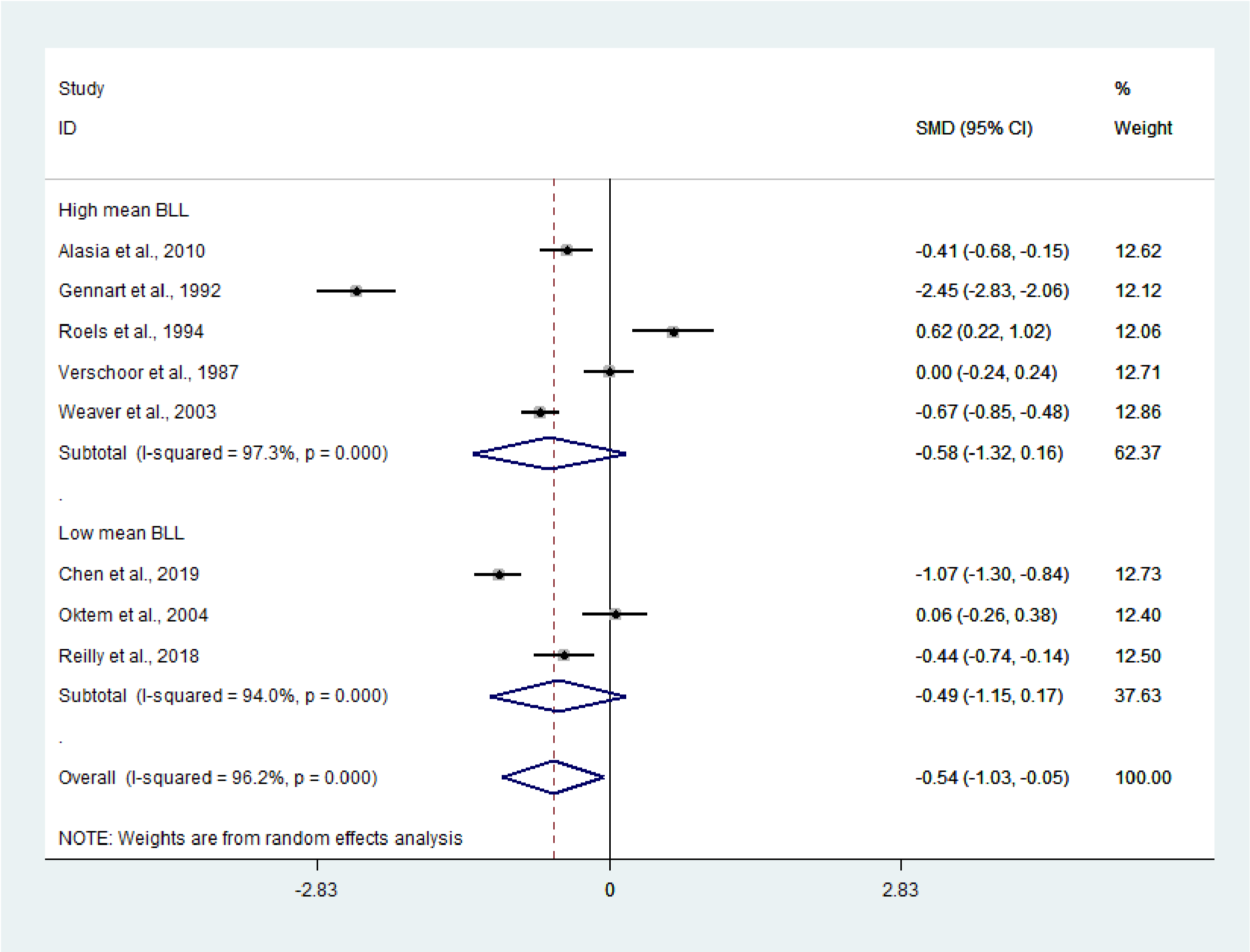

Supplementary figure 2. The difference in the mean creatinine clearance in the exposed participants was lower than that in the non-exposed participants. WMD: Weighted Mean Difference (μg/dL), % Weighted: the impact proportion of each study to the pooled effect, CI: Confidence Interval (μg/dL), Black diamond symbol: point estimate for each study, White diamond symbol: pooled WMD in each subgroup or all groups, Solid line in the middle of the graph at 0: no difference in WMD between the two groups, Dashed line: pooled WMD between the two groups

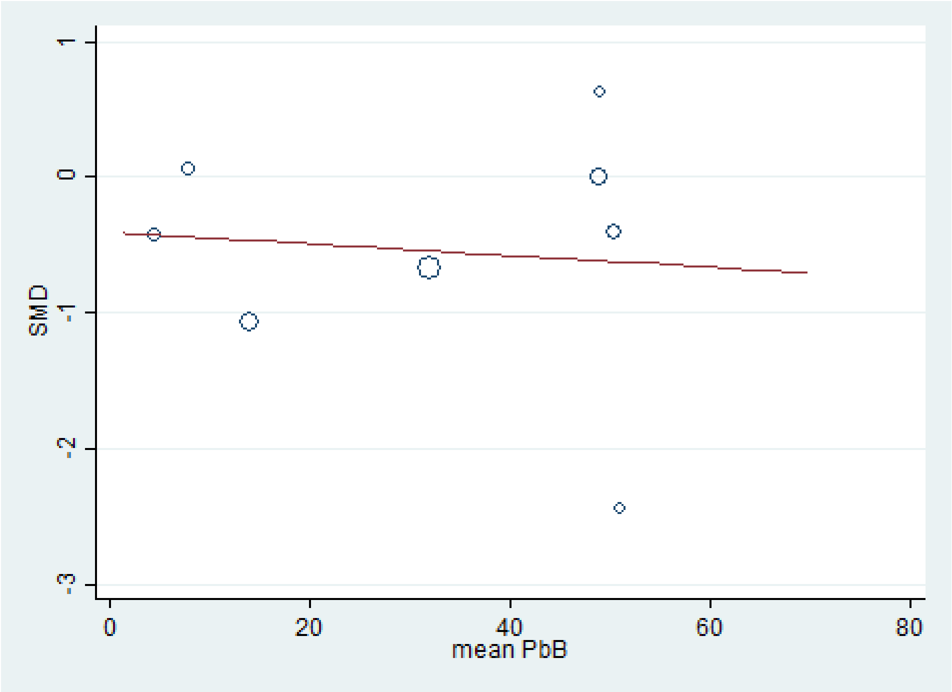

Supplementary figure 3. The meta-regression analysis of SMD (creatinine clearance) and BLL. SMD: Standardized Mean Difference, PbB: Blood Lead (μg/dL)

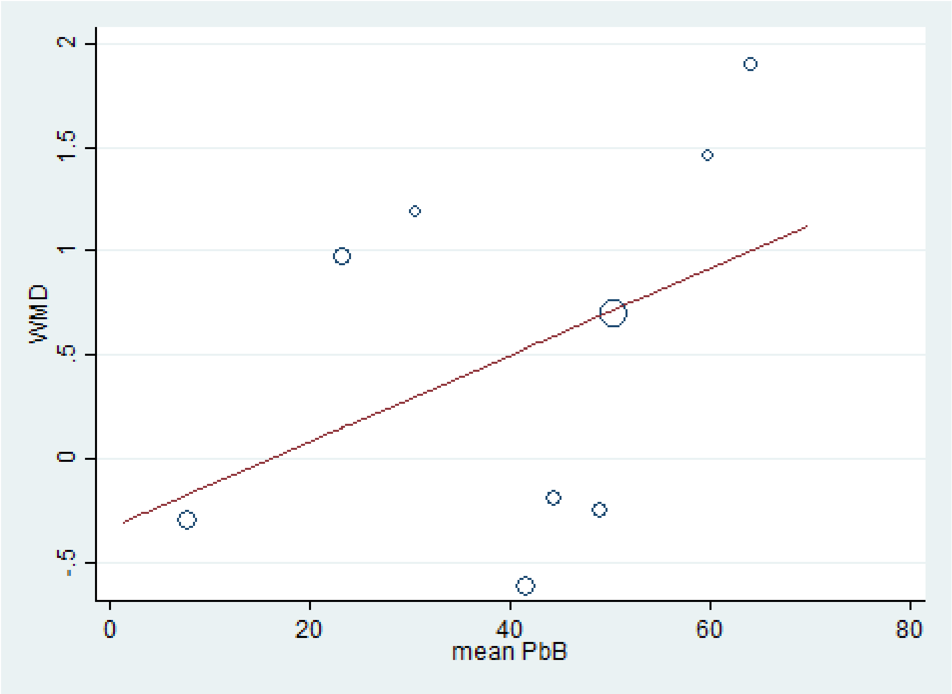

Supplementary figure 4. The meta-regression analysis of WMD (uric acid) and BLL (μg/dL)**.** WMD: Weighted Mean Difference, PbB: Blood Lead (μg/dL)
