# Supplementary material for: Blood Lead Level and Renal Impairment among Adults: A Meta-Analysis
Source: Int J Environ Res Public Health. 2021 Apr 15;18(8):4174. doi: 10.3390/ijerph18084174 (PMC8071292; doi:10.3390/ijerph18084174)
Supplement: Supplementary file 1 [file ijerph-18-04174-s001.zip › ijerph-1156141(XML)/Table S1. Search terms.pdf]

Blood Lead Level and Renal Impairment among Adults: A Meta-Analysis

Saruda Kuraeiad [1], Manas Kotepui [1*]

[1] Medical Technology, School of Allied Health Sciences, Walailak University, Tha Sala, Nakhon Si Thammarat, Thailand

Authors' Email Addresses:

[*]**Corresponding Author**: Manas Kotepui; manaskote@gmail.com

Saruda Kuraeiad; saruda.ku@wu.ac.th

S1 Table. Search term

Databases	Search terms	Search date
MEDLINE	"blood lead" OR "lead exposure" OR "lead toxicity" OR "lead poisoning") AND (renal OR kidney) AND (adult OR "middle aged" OR worker)	15 January 2021
Scopus	"blood lead" OR "lead exposure" OR "lead toxicity" OR "lead poisoning") AND (renal OR kidney) AND (adult OR "middle aged" OR worker)	15 January 2021
ISI Web of Science	"blood lead" OR "lead exposure" OR "lead toxicity" OR "lead poisoning") AND (renal OR kidney) AND (adult OR "middle aged" OR worker)	15 January 2021
