# Supplementary material for: Blood Lead Level and Renal Impairment among Adults: A Meta-Analysis
Source: Int J Environ Res Public Health. 2021 Apr 15;18(8):4174. doi: 10.3390/ijerph18084174 (PMC8071292; doi:10.3390/ijerph18084174)
Supplement: Supplementary file 1 [file ijerph-18-04174-s001.zip › ijerph-1156141(XML)/Table S2. Quality of the included studies.pdf]

Blood Lead Level and Renal Impairment among Adults: A Meta-Analysis

Saruda Kuraeiad [1], Manas Kotepui [1*]

[1] Medical Technology, School of Allied Health Sciences, Walailak University, Tha Sala, Nakhon Si Thammarat, Thailand

Authors' Email Addresses:

[*]**Corresponding Author**: Manas Kotepui; manaskote@gmail.com

Saruda Kuraeiad; saruda.ku@wu.ac.th

Table S2. Quality of the included studies

No.	Author, year, reference number	Selection				Compatibility	Exposure			Total score (8)	Rating (High, moderate, low quality)
		Is the Case Definition Adequate?	Representativeness of the Cases	Selection of Controls	Definition of Controls		Ascertainment of Exposure	Same method of ascertainment for cases and controls	Non-Response Rate		
1.	Alasia et al., 2010	❉	❉	❉	❉	❉❉	❉	❉	NA	8	High
2	Buser et al., 2016	❉	❉	NA	NA	NA	❉	NA	NA	3	Low
3.	Chen et al., 2019	❉	❉	❉	❉	❉❉	❉	❉	NA	8	High
4.	Chung et al., 2013	❉	❉	NA	NA	NA	❉	NA	NA	3	Low
5.	de Pinto Almeida et al., 1987	❉	❉	❉	❉	❉❉	❉	❉	NA	8	High
6.	Dioka et al., 2004	❉	❉	❉	❉	❉❉	❉	❉	NA	8	High

7.	Ehrlich et al., 1998	❋	❋	NA	NA	NA	❋	NA	NA	3	Low
8.	Gennart et al., 1992	❋	❋	❋	❋	❋❋	❋	❋	NA	8	High
9.	Gerhardsson et al., 1998	❋	❋	❋	❋	❋❋	❋	❋	NA	8	High
10.	Gerhardsson et al., 1992	❋	❋	NA	NA	NA	❋	NA	NA	3	Low
11.	Goswami et al., 2001	❋	❋	NA	NA	NA	❋	NA	NA	3	Low
12.	Harar et al., 2018	❋	❋	NA	NA	NA	❋	NA	NA	3	Low
13.	Herna´ndez-Serrato et al., 2006	❋	❋	NA	NA	NA	❋	NA	NA	3	Low
14.	Jain RB, 2019	❋	❋	NA	NA	NA	❋	NA	NA	3	Low
15.	Jung et al., 1998	❋	❋	❋	❋	❋❋	❋	❋	NA	8	High
16.	Kim et al., 1996	❋	❋	NA	NA	NA	❋	NA	NA	3	Low
17.	Kshirsagar et al., 2020	❋	❋	❋	❋	❋❋	❋	❋	NA	8	High
18	Kshirsagar et al., 2019	❋	❋	❋	❋	❋❋	❋	❋	NA	8	High
19.	Lai et al., 2008	❋	❋	NA	NA	NA	❋	NA	NA	3	Low
20.	Lim et al., 2001	❋	❋	NA	NA	NA	❋	NA	NA	3	Low
21.	Lin et al., 2007	❋	❋	❋	❋	❋❋	❋	❋	NA	8	High
22.	Lu et al., 2015	❋	❋	NA	NA	NA	❋	NA	NA	3	Low
23.	Mujaj et al., 2019	❋	❋	NA	NA	NA	❋	NA	NA	3	Low
24.	Muntner et al., 2003	❋	❋	NA	NA	NA	❋	NA	NA	3	Low
25.	Nakhaee et al., 2018	❋	❋	NA	NA	NA	❋	NA	NA	3	Low
26.	Navas-Acien et al., 2009	❋	❋	NA	NA	NA	❋	NA	NA	3	Low
27.	Oktem et al., 2004	❋	❋	❋	❋	❋❋	❋	❋	NA	8	High

28.	Omae et al., 1990	❉	❉	NA	NA	NA	❉	NA	NA	3	Low
29.	Onuegbu et al., 2011	❉	❉	❉	❉	❉❉	❉	❉	NA	8	High
30.	Patil et al., 2007	❉	❉	❉	❉	❉❉	❉	❉	NA	8	High
31.	Payton et al., 1994	❉	❉	NA	NA	NA	❉	NA	NA	3	Low
32.	Reilly et al., 2018	❉	❉	❉	❉	❉❉	❉	❉	NA	8	High
33.	Roels et al., 1994	❉	❉	❉	❉	❉❉	❉	❉	NA	8	High
34.	Satarug et al., 2004	❉	❉	NA	NA	NA	❉	NA	NA	3	Low
35.	Staessen et al., 1990	❉	❉	NA	NA	NA	❉	NA	NA	3	Low
36.	Staessen et al., 1992	❉	❉	NA	NA	NA	❉	NA	NA	3	Low
37.	Tsaih et al., 2004	❉	❉	NA	NA	NA	❉	NA	NA	3	Low
38.	Verschoor et al., 1987	❉	❉	❉	❉	❉❉	❉	❉	NA	8	High
39.	Wang et al., 2002	❉	❉	NA	NA	NA	❉	NA	NA	3	Low
40.	Wang et al., 2018	❉	❉	NA	NA	NA	❉	NA	NA	3	Low
41.	Weaver et al., 2011	❉	❉	NA	NA	NA	❉	NA	NA	3	Low
42.	Weaver et al., 2003	❉	❉	NA	NA	NA	❉	NA	NA	3	Low
43.	Weaver et al., 2005	❉	❉	❉	❉	❉❉	❉	❉	NA	8	High

❉ A star rating
